# Fractionated free fatty acids and their relation to diabetes status after Roux‐en‐Y gastric bypass: A cohort study

**DOI:** 10.14814/phy2.14708

**Published:** 2021-01-19

**Authors:** Freja Eriksen, Elin R. Carlsson, Jens K. Munk, Sten Madsbad, Mogens Fenger

**Affiliations:** ^1^ Department of Clinical Biochemistry Copenhagen University Hospital Hvidovre Hvidovre Denmark; ^2^ Department of Clinical Biochemistry Nordsjaellands Hospital University of Copenhagen Hillerod Denmark; ^3^ Department of Endocrinology Copenhagen University Hospital Hvidovre Hvidovre Denmark

**Keywords:** free fatty acids, gas chromatography–mass spectrometry, Roux‐en‐Y Gastric Bypass, type 2 diabetes

## Abstract

Bariatric surgery is associated with near‐immediate remission of type 2 diabetes and recently suggested as a treatment for type 2 diabetes. Specifically, Roux‐en‐Y gastric bypass has been a focus of much research, but still, the mechanisms of action are only partly elucidated. We aim to investigate whether some mechanisms might be mediated by free fatty acids (FFAs). We measured eight fractionated FFAs before and up to 2 years after Roux‐en‐Y gastric bypass surgery in 207 patients, divided into three groups. One non‐diabetic group, one diabetic group with post‐operative remission and one diabetic group with persistent diabetes after surgery. Pre‐ and postoperative levels of fractionated FFAs were compared within and between groups. The sum of the measured FFAs were lower in the group with persistent diabetes, compared to the other groups. The pre‐surgery level of linoleic acid in the group with persistent diabetes was significantly lower compared to the other two groups. The levels of fractionated FFAs decreased from pre‐surgery to three months after surgery, except for oleic acid and arachidonic acid and for Docosahexaenoic acid (DHA) in the non‐diabetic group. The FFAs with decreasing levels from pre‐surgery to three months post‐surgery are all precursors to oleic acid, arachidonic acid, and DHA, respectively, which may imply a drift, indicating that they need to be sustained at an acceptable level for optimal metabolic function. The fact that the sum of the measured FFAs is lower in the group with persistent diabetes may suggest that this group and the group with diabetes remission represent two distinct types of type 2 diabetes. It is proposed that linoleic acid could be used as a biomarker to determine the plausibility for type 2 diabetes remission after Roux‐en‐Y gastric bypass surgery.

## Graphical abstract

We aim to investigate whether some mechanisms regarding remission of type 2 diabetes after Roux‐en‐Y gastric bypass might be mediated by free fatty acids (FFAs). Pre‐ and postoperative levels of fractionated FFAs were compared within and between a non‐diabetes group and two diabetes groups with and without remission respectively. The sum of the measured FFAs is lower in the group with persistent diabetes which may suggest that this group and the group with diabetes remission represent two distinct types of type 2 diabetes.

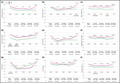

## 1 INTRODUCTION

Roux‐en‐Y gastric bypass (RYGB) surgery is a well‐known treatment for obesity, and research has shown that RYGB often simultaneously leads to remission of type 2 diabetes, even before significant weight loss (Borgeraas et al., 2020). When compared to intensive medical therapy and lifestyle intervention, recent evidence suggests that metabolic surgery is the most effective treatment for patients with type 2 diabetes (Pérez‐pevida et al., 2019) in improving glycemic control and in reducing mortality (Sjöström, 2013). It has been shown that hepatic insulin sensitivity improves as early as one week after RYGB (Bojsen‐Møller et al., 2014, 2017). Later after months and major weight loss insulin sensitivity in the skeletal muscles and fat tissue is also improved (Bojsen‐Møller et al., 2014). Although some patients relapse to type 2 diabetes postoperatively, the remission and protection from type 2 diabetes are sustained over long periods of time as well, even after considerable weight regain (Hoffstedt et al., 2017).

Adipose tissue insulin resistance is characterized by defective insulin‐mediated glucose transport, a decreased capacity for lipid uptake, and a failure to suppress lipolysis and inflammation, resulting in elevated plasma free fatty acids (FFA) and cytokines (Javeed & Matveyenko, 2018). This aberrant adipose tissue metabolism in type 2 diabetes directly contributes to insulin resistance in target tissues through an increase in ectopic lipid accumulation or indirectly through the cytokine‐mediated disruption of the insulin signaling cascade in the liver and skeletal muscle (Javeed & Matveyenko, 2018).

The major component of lipids are fatty acids, and the physical, chemical, and physiological properties of a lipid class depend primarily on its fatty acids composition (Ichihara & Fukubayashi, 2010). Fatty acids are either saturated or unsaturated carboxylic acids with carbon chains varying between 2 and 36 carbon atoms.

**FIGURE 1:**
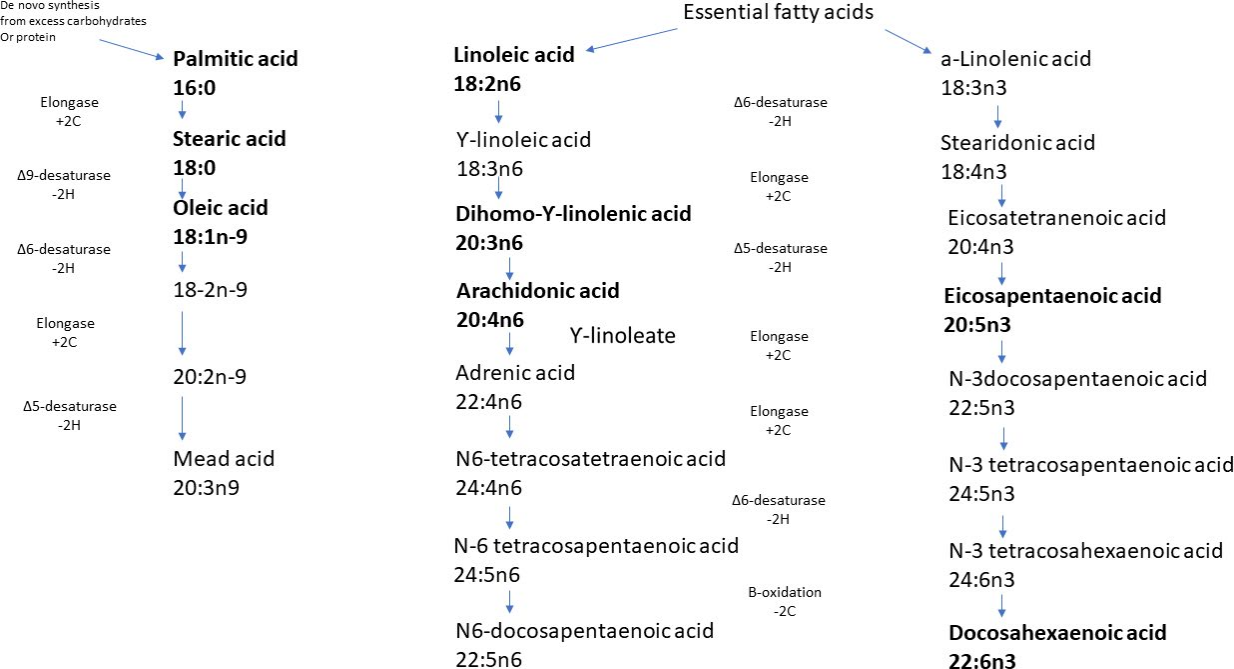
1An overview of the eight measured FFAs (written in bold letters) and their pathways. The first number in the chemical formula represents the amount of carbon atoms in the fatty acid carbon chain. The second number refers to the amount of double bonds in the fatty acid. The number after the n indicates where the double bond closest to the methyl end of the fatty acid chain is located (Stankova et al., 2011)

As shown in Figure 1, the FFAs measured in this study are metabolized through three distinct pathways: 1) The saturated fatty acid palmitic acid is converted to stearic acid by the action of elongase which in turn is converted to the monounsaturated oleic acid by the action of delta‐9‐desaturase; 2) In the omega 6 pathway, the essential linoleic acid is converted to dihomo‐γ‐linolenic acid (DGLA) by the action of elongase and delta‐6‐desaturase and DGLA is converted to arachidonic acid by the action of delta‐5‐desaturase; 3) In the omega 3 pathway, the essential fatty acid alpha‐linolenic acid is converted to eicosapentaenoic acid (EPA) by the action of delta‐6‐desaturase, elongase, and delta‐5‐desaturase. EPA is converted to docosahexaenoic acid (DHA) by the action of elongase, delta‐6‐desaturase, and beta‐oxidation (Das, 2006). In mammals there is no cross metabolization between the three pathways: 1) the palmitic acid‐stearic acid‐oleic acid pathway; 2) the omega‐6 pathway; and 3) the omega 3 pathway (Burdge, 2019).

FFAs are integrated into phospholipids in the cell membrane where phospholipase induces the release of DGLA, arachidonic acid, EPA, and DHA to serve as precursors to their respective metabolites. Since the source of these metabolites plays a major role in physiological functions, an imbalance in the combination of FFAs in the cell membrane could lead to increased inflammation and decreased insulin sensitivity (Shahidi, 2018). Decreased fluidity of the membrane by an increased content of saturated FFA content in phospholipids leads to a decrease in the number of insulin receptors and the affinity of insulin to them. In contrast, the presence of polyunsaturated fatty acids in plasma membrane phospholipids increases its fluidity and has been associated with improved insulin sensitivity (Perona, 2017).

It has been proposed that different types of FFAs may exert a differential effect on specific receptors associated with insulin resistance (Sobczak & Blindauer, 2019). Thus, the ratio between levels of specific FFAs might be important for the development of insulin resistance.

After RYGB, studies have shown a general decrease in FFAs in patients with remission of type 2 diabetes (Carswell et al., 2016). However, only a few studies have investigated the impact of RYGB on FFA levels and only three of them report data on fractionated FFAs (Luo et al., 2016; Nemati et al., 2017; Thomas et al., 2016). As far as we know only one study has investigated the long‐term impact of RYGB on fractionated FFAs (Luo et al., 2016). In this study, we aim to investigate whether some mechanisms regarding RYGB mediated diabetes remission might be mediated by FFAs.

## 2 DESIGN OF STUDY AND METHODS

The research population consists of patients treated for obesity between 2009 and 2014 at Copenhagen University Hospital in the Capital Region of Denmark and has been previously described (Fenger et al., 2016). From this population, we selected all patients that underwent RYGB surgery between November 2010 and September 2013, who had delivered a fasting plasma blood sample before their operation, and in addition a fasting sample within 4 months post‐surgery. We excluded a heterogeneous group of 15 patients with possible type 2 diabetes in treatment with diet or anti‐diabetic medicine before surgery, but with no confirmed hyperglycemia in lab data and off antidiabetic treatment after RYGB and one patient who had type 2 diabetes and hyperglycemia before RYGB in biochemical remission but still on antidiabetic treatment after RYGB. We included all fasting plasma samples from the selected population up to 2 years after surgery, ending up with a total of 795 samples from 207 patients. As previously described samples were divided into duration after surgery: 3 (n = 207), 6 (n = 149), 12 (n = 138), and 24 (n = 94) months after RYGB (Carlsson et al., 2018). Samples were frozen at −80°C and stored between 5 and 8 years at the time for the measurement of plasma fractionated free fatty acids. The surgical technique is described in (Fenger et al., 2016).

The clustering of the research population in groups according to diabetes status before and after surgery has been previously described (Carlsson et al., 2018). Three groups are addressed in this paper; NDM, a non‐diabetes group (n = 153); DMH‐NDM, a group of patients with type 2 diabetes and hyperglycemia with post‐surgery diabetes remission (n = 34); and DMH‐DMH, a group of patients with type 2 diabetes and hyperglycemia with persistent diabetes after RYGB (n = 20). Remission was defined as a decrease in HbA_1c_ to below 48 mmol/mol (6.5%) without antidiabetic medication for as long as clinical and laboratory data were available, varying from 2 years to a maximum of 5 years.

After surgery, patients were instructed to eat according to general international dietary recommendations and common practice after RYGB (Dagan et al., 2017; Nordic Counsil of Ministers, 2008). From month four and onwards, the dietary plan was individualized by a clinical dietician to meet the specific needs of each patient. One year after surgery when weight was stabilized, the recommended daily calorie intake corresponded to energy expenditure for the individual patient.

This study was performed in accordance with the Helsinki Declaration and was approved by the Scientific Ethics Committee of the Capital Region, Denmark, protocol number HD2009‐78, extended with the protocol number H‐6‐2014‐029, and by the Danish Data Protection Agency. Informed consent was obtained in writing from all the participants in this study.

### 2.1 Data and resource availability

All data generated or analyzed during this study are included in the published article (and its online supplementary files). No applicable resources were generated or analyzed during the current study.

### 2.2 FREE FATTY ACID SAMPLE PREPARATION AND ANALYSIS

The Gas Chromatography‐Mass Spectrometry method we used to measure FFA levels was able to stably quantify eight FFAs: C16:0 (palmitic acid), C18:0 (stearic acid), C18:1n‐9 (oleic acid), C18:2n‐6 (linoleic acid), C20:3n‐6 (dihomo‐γ‐linolenic acid, DGLA), C20:4n‐6 (arachidonic acid), C20:5n‐3 (eicosapentaenoic acid, EPA) and C22:6n‐3 (docosahexaenoic acid, DHA).

We aimed to measure non‐esterified fatty acids (NEFAs), non‐esterified fatty acids (EFAs), that is, the EFAs must be removed from the samples. The method was modified from previously described methods (Han et al., 2011; Ichihara & Fukubayashi, 2010). To the plasma samples (100 µl) 100 µl of a premade mixture of 0.4 M potassium hydroxide (KOH) in methanol and 5 µl of C17 internal standard (a fatty acid which is not produced in humans) in methanol were added. The mixture was vortexed and stabilized at room temperature for 3 min. Hexane (1 ml) was added and vortexed to obtain the separation of the polar FFAs from the EFAs. After phase separation, the upper layer containing EFAs was removed. To the bottom layer containing the NEFAs 100 ml of 5% sulfuric acid in methanol was added to methylate the NEFAs. The vials were sealed and incubated at 70°C for 30 min to complete methylation. Sterile water (400 µl) was added to quench the derivatization and 100 µl of hexane was added for phase separation. The upper layer (600 µl) containing the now methylated NEFAs were recovered to new vials and dried under a stream of nitrogen. The methylated FFA was finally dissolved in 200 µl hexane.

The fatty acid methyl esters were separated by gas–liquid chromatography (Shimadzu, Japan) on an Omegawax®250 Capillary column, 30 m × 0.25 mm × 0.25 µm film thickness and with helium as a carrier gas. The temperature gradient was programmed to 200–280°C. The FFAs were identified, by comparing the retention time of each peak with the retention time of standards of individual fatty acid methyl esters (Sigma‐Aldrich, minimum 99% purity). The concentration was determined using quadratic regression equations obtained from a five‐point calibration curve, specific for each standard representing an individual FFA. All curves had high correlation coefficients (r > 0.996), indicating that our data fits well with the chosen regression.

We did not measure total FFA, so when the term total FFA is used throughout the article it represents the sum of the eight FFAs.

### 2.3 Statistical analysis

One‐way ANOVA and Tukey post hoc test were used to compare: FFA levels and preoperative clinical characteristics between groups; weight, BMI and weight loss at 3‐, 6‐, 12‐, and 24‐months post‐surgery; FFA levels and ratios at specific time points between groups. To compare pre‐surgery FFA levels and specific FFA ratios, with post‐surgery FFA levels and ratios within the same patient group, we used paired t‐test. Unpaired t‐test was used to investigate whether either gender or statin treatment had an effect on pre‐surgery FFA levels.

Normal distribution and homogeneity of variances of most parameters allowed for use of parametric tests. Where Levene's test of equality of variances was statistically significant, the one‐way ANOVA was performed with a Welch‐Satterthwaite correction followed by a Games‐Howell post hoc test. For a few parameters without normal distribution, significant differences between groups or between post‐ and pre‐surgery values in the same group were found using the one‐way ANOVA or paired *t*‐test. In these instances, the significance was confirmed with a Kruskal–Wallis *H*‐test or Wilcoxon signed‐rank test. The highest *p*‐value was used to avoid overestimating the significance. When the p‐value from Kruskal–Wallis *H*‐test was used, multiple Mann–Whitney *U* tests were performed using the Bonferroni correction to determine the subgroups with a significant difference.

Nominal *p*‐values lower than 0.05 were considered statistically significant. Throughout the article, data are written as mean with a 95% confidence interval. The IBM SPSS version 25 was used for all analyses.

## 3 RESULTS

### 3.1 Pre‐surgery clinical characteristics

The preoperative clinical characteristics for all patients and in the three groups are shown in Table 1. Notably, in the group of patients with diabetes, there was a lower female to male ratio, higher age, and lower total‐ and LDL cholesterol levels compared to the group without diabetes.

**TABLE 1:** Preoperative clinical characteristics for all patients and patients grouped according to diabetes status

	All patients (n = 207) Mean (SD)	NDM (n = 153) Mean (SD)	DMH – NDM (n = 34) Mean (SD)	DMH – DMH (n = 20) Mean (SD)	ANOVA *p*‐value
Age (years)	44.3 (9.5)	42.0 (9.0)	50.4 (8.2)*	51.5 (7.4)*	3 × 10^−8^
Gender (f/m)	142/65	115/38	18/16*	9/11*	0.002
Height (cm)	171.6 (9.8)	171.1 (9.9)	174.7 (8.1)	171.2 (11.4)	0.155
Weight (kg)	125.8 (21.8)	126.8 (22.3)	126.2 (20.0)	117.2 (19.7)	0.194
BMI (kg/m^2^)	42.6 (5.7)	43.1 (5.9)	41.6 (5.26)	40.0 (3.6)*	0.050
Systolic BP (mmHg)	127.8 (14.7)	126.9 (15.0)	131.2 (12.8)	128.2 (14.7)	0.310
Diastolic BP (mmHg)	82.0 (10.3)	82.0 (11.1)	81.6 (6.8)	82.4 (10.1)	0.958
HbA_1c_ (mmol/mol)	39.3 (10.9) (n = 197)	34.5 (3.8) (n = 144)	50.0 (12.0)*	56.4 (13.9)*	1 × 10^−10^
Total cholesterol (mmol/L)	4.78 (1.06) (n = 196)	4.98 (0.96) (n = 144)	4.25 (1.1)*	4.24 (1.1)*	8 × 10^−5^
HDL‐cholesterol (mmol/L)	1.16 (0.31) (n = 196)	1.20 (0.29) (n = 144)	1.05 (0.36)*	1.09 (0.35)	0.028
LDL‐ cholesterol (mmol/L)	2.91 (0.9) (n = 192)	3.11 (0.85) (n = 143)	2.38 (0.98)* (n = 31)	2.27 (1.1)* (n = 18)	3 × 10^−6^
VLDL‐ cholesterol (mmol/L)	0.71 (0.33) (n = 192)	0.672 (0.30) (n = 143)	0.765 (0.30) (n = 31)	0.889 (0.47) (n = 18)	0.080
Triglycerides (mmol/L)	1.68 (1.12) (n = 196)	1.51 (0.74) (n = 144)	2.15 (2.0)	2.13 (1.3)	0.038
Statins(+/‐)	56/146 (n = 202)	20/129 (n = 149)	21/12* (n = 33)	15/5*	2 × 10^−8^
Total fatty acids mmol/L	4.73 (1.09)	4.73 (1.09)	4,97 (1.31)	4.30 (1.13)	0.113
Palmitic acid mmol/L	1.74 (0.47)	1.74 (0.46)	1.83 (0.55)	1.59 (0.46)	0.209
Stearic acid mmol/L	0.531 (0.12)	0.533 (0.12)	0.531 (0.13)	0.496 (0.10)	0.407
Oleic acid mmol/L	0.868 (0.24)	0.941 (0.23)	0.873 (0.25)	0.809 (0.32)	0.103
Linoleic acid mmol/L	0.857 (0.27)	0.875 (0.25)	0.873 (0.33)	0.692 (0.25)**	0.014
DGLA mmol/L	0.0968 (0.040)	0.0970 (0.039)	0.0837 (0.049)	0.0968 (0.028)	0.310
Arachidonic acid mmol/L	0.470 (0.17)	0.465 (0.16)	0.505 (0.20)	0.454 (0.17)	0.420
EPA mmol/L	0.0425 (0.024)	0.0400 (0.023)	0.0532 (0.030)*	0.048 (0.023)	0.011
DHA mmol/L	0.125 (0.049)	0.123 (0.046)	0.138 (0.058)	0.123 (0.056)	0.259

Data are reported as mean (SD). If the number of patients with available clinical data was less than 95% of the total of patients in the group, the actual number is specified. SD, Standard deviation; HbA_1c_, Glycated hemoglobin; HDL‐ cholesterol, High‐density lipoprotein cholesterol; LDL‐cholesterol, Low‐density lipoprotein cholesterol; VLDL‐cholesterol, Very low‐density lipoprotein cholesterol; NDM, Patients without diabetes mellitus (DM); DMH‐NDM, Patients with DM in remission after Roux‐en‐Y gastric bypass surgery (RYGB); DMH‐DMH, Patients with DM not in remission after RYGB. *p*‐value is from One‐way ANOVA comparing the three patient‐subgroup means; * indicates significant difference (*p* < 0.05) when compared to the NDM group. ** indicates significant difference compared to both the NDM group and the DMH – NDM group. Post hoc p‐values from Tukey and Games‐Howell are not shown in table.

Body weight and BMI were similar in all three subgroups before surgery (mean BMI 42.4 (41.7–43.1) kg/m^2^) as well as at all time points after RYGB (24 months BMI: 29.7 (28.3–31.2) kg/m^2^), which is shown in more detail in the supplementary material, Table S1, together with data for absolute and relative weight loss.

Statin treatment was more frequent in the two subgroups with diabetes patients compared to the subgroup without diabetes.

The plasma concentration of linoleic acid was significantly lower for the group with persistent diabetes compared to the other two subgroups, while EPA was significantly higher in the group with diabetes remission compared to the non‐diabetes group. EPA was also increased in the group with persistent diabetes but did not reach statistical significance.

The only parameter with a significant difference between the two diabetes groups was linoleic acid which showed a lower level in the group with persistent diabetes compared to the group with diabetes remission.

When analyzing pre‐surgery levels of FFAs for all patients together, we found that linoleic acid was higher in female patients compared to male patients (see supplementary, Table S2, *p* = 0.018) and that patients receiving statin treatment had lower levels of linoleic acid and higher levels of EPA compared to patients receiving no lipid‐lowering medicine (see supplementary, Table S3). Furthermore, linoleic acid was significantly lower in the patients receiving statin treatment compared to the patients with no lipid‐lowering medicine in the DMH‐NDM group.

### 3.2 Total and individual FFA levels

**FIGURE 2:**
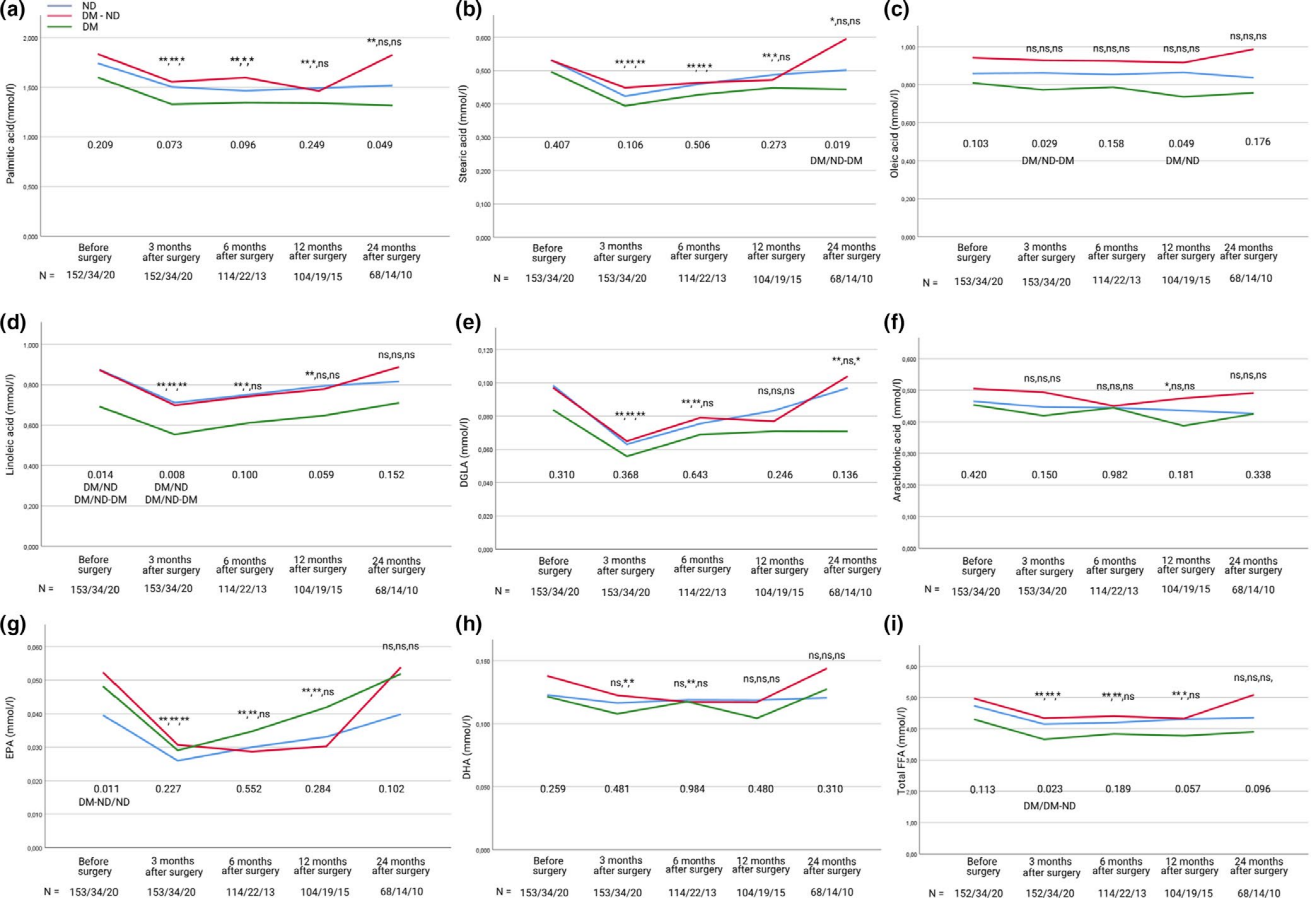
Changes in palmitic acid (a), Stearic acid (b), Oleic acid (c), Linoleic acid (d), DGLA (Dihomo‐Ƴ‐linolenic acid) (e), arachidonic acid (f), EPA (Eicosapentaenoic acid) (g), DHA (Docosahexaenoic acid) (h) and total FFA (i) (The eight FFAs added together) at 3, 6, 12, or 24 months after Roux‐en‐Y gastric bypass (RYGB) surgery in three patient subgroups: ND, patients without diabetes mellitus (DM) (blue); DM‐ND, patients with DM in remission after RYGB (red) and DM‐DM, patients with DM and continued hyperglycemia after RYGB (green). Data are shown as mean with error bars representing a 95% confidence interval of the mean. Significant different values compared with corresponding preoperative value are shown as * (*p* < 0.05), ** (*p* < 0.01) and ns (not significant) in the order ND, DM‐ND, DM‐DM. P‐values from one‐way ANOVA comparing the three groups at specific time points are located underneath each curve. With post hoc testing significant difference was found between the groups stated beneath p‐values. The number of patients (N) for whom we have data at each time point are shown in the order ND/DM‐ND/DM‐DM at the bottom of each figure

Compared to pre‐surgery levels, total FFA levels were significantly lower at 3 months post‐surgery in all patient groups (Table 2). Total FFA levels were still significantly lower at 6 months post‐surgery for the group with diabetes remission and at 12 months for the group without diabetes (Table 2). For the group with persistent diabetes, no significant difference was found after 6 months. Figure 2I shows that the group with persistent diabetes has lower levels of total FFA compared to the other two groups, but with the only significant difference at 3 months post‐surgery (for absolute values see supplementary material Table S4). Total FFA levels returned to pre‐surgery levels in all three groups after 24 months.

**TABLE 2:** Total FFA delta values (pre‐surgery values minus post‐surgery values) and paired T‐tests grouped according to diabetes status and ANOVA

mmol/L	All		NDM		DMH‐NDM		DMH – DMH		ANOVA
	Mean delta (95% CI)	Paired *t*‐test *p*‐value	Mean delta (95% CI)	Paired *t*‐test *p*‐value	Mean delta (95% CI)	Paired T‐test	Mean delta (95% CI)	Paired *t*‐test *p*‐value	*p*‐value
3 months									
Total FFA	0.594 (0.470–0.718)	1 × 10^−19^	0.580 (0.442–0.717)	5 × 10^−14^	0.631 (0.270–0.993)	0.001	0.642 (0.146–1.137)	0.014**	0.023
6 months									
Total FFA	0.581 (0.427–0.734)	6 × 10^12^	0.534 (0.386–0.693)	2 × 10^−10^	0.762 (0.208–1.317)	0.009	0.636 (−0.187–1.458)	0.118	0.189
12 months									
Total FFA	0.473 (0.305–0.640)	1 × 10^−7^	0.452 (0.269–0.634)	4 × 10^−6^	0.595 (0.054–1.136)	0.33	0.461 (−0.227–1.150)	0.173*	0.057
24 months									
Total FFA	0.199 (−0.020–0.418)	0.074	0.218 (−0.002–0.430)	0.052	−0.152 (−0.978–0.674)	0.697	0.557 (−0.405–1.519)	0.223	0.096

Data are reported as mean (SD). SD, Standard deviation; FFA, Free fatty acids; NDM, Patients without diabetes mellitus; DMH‐NDM, Patients with DM in remission after Roux‐en‐Y gastric bypass surgery (RYGB); DMH‐DMH, Patients with DM not in remission after RYGB. One‐way ANOVA (or Kruskal–Wallis H‐test) is comparing the three patient‐subgroup means (absolute mean, not delta) at either 3, 6, 12, or 24 months; * indicates significant difference (*p* < 0.05) when compared to the NDM group; ** indicates significant difference compared to the DMH‐NDM group. Values from Mann–Whitney U‐test and post hoc p‐values from Tukey and Games‐Howell are not shown in table.

In the group without diabetes, palmitic acid and stearic acid decreased significantly post‐surgery and stayed at a significantly lower level until 24 months post‐surgery. Palmitic acid and stearic acid levels normalized after 24 months in the group with diabetes remission whereas they normalized after 6 and 12 months, respectively, in the group with persistent diabetes. In contrast, oleic acid was kept at a consistent level with no significant differences in any of the groups at any time point (Table 3). Figure 2 (A,B,C) shows that FFA levels in the group with persistent diabetes are lower compared to the other groups. However, the only significant difference between groups was found at 3‐ and 12 months post‐surgery for oleic acid and at 24 months post‐surgery for palmitic acid and stearic acid. At 24 months post‐surgery palmitic acid and stearic acid levels are higher in the group with diabetes remission compared to the group with persistent diabetes. However, the difference was not significant for palmitic acid after post hoc testing (for absolute values see supplementary material Table S4).

**TABLE 3:** Palmitic acid, stearic acid, and oleic acid delta values (pre‐surgery values minus post‐surgery values) and paired T‐tests grouped according to diabetes status and ANOVA

mmol/L	All		NDM		DMH‐NDM		DMH – DMH		ANOVA
	Mean delta (95% CI)	Paired *t*‐test *p*‐value	Mean delta (95% CI)	Paired *t*‐test *p*‐value	Mean delta (95% CI)	Paired *t*‐test *p*‐value	Mean delta (95% CI)	Paired *t*‐test *p*‐value	*p*‐value
3 months									
Palmitic acid	0.248 (0.191–0.304)	2 × 10^−15^	0.237 (0.178–0.297)	6 × 10^−13^	0.281 (0.063–0.575)	0.003	0.270 (0.019–0.520)	0.036	0.073
Stearic acid	0.103 (0.089–0.118)	8 × 10^−33^	0.108 (0.092–0.125)	5 × 10^−26^	0.082 (0.047–0.117)	3 × 10^−5^	0.102 (0.052–0.151)	4 × 10^−4^	0.106
Oleic acid	0.004 (−0.025–0.032)	0.796	−0.003 (−0.034–0.028)	0.864	0.013 (−0.061–0.087)	0.715	0.036 (−0.092–0.165)	0.561**	0.029
6 months									
Palmitic acid	0.286 (0.219–0.353)	2 × 10^−15^	0.275 (0.208–0.32)	5 × 10^−13^	0.319 (0.063–0.574)	0.017	0.331 (0.002–0.661)	0.131	0.096
Stearic acid	0.080 (0.063–0.097)	2 × 10^−16^	0.080 (0.061–0.098)	1 × 10^−13^	0.088 (0.030–0.146)	0.005	0.064 (0.002–0.126)	0.043	0.506
Oleic acid	0.021 (−0.013–0.056)	0.227	0.008 (−0.025–0.041)	0.623	0.041 (−0.073–0.155)	0.461	0.100 (−0.119–0.319)	0.340	0.158
12 months									
Palmitic acid	0.276 (0.200–0.353)	6 × 10^−11^	0.274 (0.188–0.360)	7 × 10^−9^	0.320 (0.080–0.559)	0.012	0.239 (−0.048–0.526)	0.096	0.249
Stearic acid	0.054 (0.037–0.071)	9 × 10^−9^	0.054 (0.034–0.075)	8 × 10^−7^	0.067 (0.013–0.121)	0.023	0.037 (−0.010–0.084)	0.113	0.273
Oleic acid	0.009 (−0.029–0.047)	0.641	−0.005 (−0.043–0.032)	0.774	0.023 (−0.088–0.134)	0.670	0.091 (−0.112–0.294)	0.355*	0.049
24 months									
Palmitic acid	0.128 (0.034–0.222)	0.008	0.128 (0.046–0.230)	0.004	−0.080 (−0.438–0.277)	0.635	0.348 (−0.059–0.756)	0.085	0.049
Stearic acid	0.014 (−0.008–0.036)	0.201	0.027 (0.002–0.052)	0.038	−0.060 (−0.127–0.007)	0.074	0.032 (−0.008–0.072)	0.105**	0.019
Oleic acid	0.006 (−0.044–0.056)	0.803	0.004 (−0.041–0.050)	0.855	−0.050 (−0.235–0.135)	0.570	0.099 (−0.189–0.385)	0.452	0.176

Data are reported as mean (SD). SD, Standard deviation; FFA, Free fatty acids; NDM, Patients without diabetes mellitus (DM); DMH‐NDM, Patients with DM in remission after Roux‐en‐Y gastric bypass surgery (RYGB); DMH‐DMH, Patients with DM not in remission after RYGB. All patients also include 15 patients who belong to other subgroups than the three shown in table. One‐way ANOVA (or Kruskal–Wallis H‐test) is comparing the three patient‐subgroup means (absolute mean, not delta) at either 3, 6, 12, or 24 months; * indicates significant difference (*p* < 0.05) when compared to the NDM group; ** indicates a significant difference compared to the DMH‐NDM group. Values from Mann–Whitney U‐test and Post hoc p‐values from Tukey and Games‐Howell are not shown in table.

As expected, for some FFAs the concentrations were very low whereas others had markedly higher concentrations. The pre‐surgery individual FFAs percentages of total FFA was as follows: palmitic acid (36,8%), stearic acid (11,2%), oleic acid (18,4%), linoleic acid (18,1%), DGLA (2,0%), arachidonic acid (9,9%), EPA (0,9%) and DHA (2,6%).

Linoleic acid and DGLA levels decreased significantly after surgery. After 6 months, no significant changes were found in the group with persistent diabetes whereas the levels of Linoleic acid and DGLA were significantly lower compared to pre‐surgery levels until 6 months in the group with diabetes remission and until 12 months in the group without diabetes. In contrast, arachidonic acid did not differ in all groups between time points (Table 4). When comparing the levels between groups, the only significant difference was found for linoleic acid at 3 months post‐surgery, and before surgery as stated earlier. The level of linoleic acid was significantly lower in the group with persistent diabetes compared to the other two groups. In Figure 2d,e, it shows how linoleic acid and DGLA has the same tendency as palmitic acid and stearic acid to increase after 24 months. However, no significant difference in linoleic acid and DGLA levels was found between the groups (for absolute values see supplementary material Table S4).

**TABLE 4:** Linoleic acid, DGLA, and arachidonic acid delta values (presurgery values minus post‐surgery values) and paired T‐tests grouped according to diabetes status and ANOVA

mmol/L	All		NDM		DMH‐NDM		DMH – DMH		ANOVA
	Mean delta (95% CI)	Paired *t*‐test *p*‐value	Mean delta (95% CI)	Paired *t*‐test *p*‐value	Mean delta (95% CI)	Paired *t*‐test *p*‐value	Mean delta (95% CI)	Paired *t*‐test *p*‐value	*p*‐value
3 months									
Linoleic acid	0.163 (0.134–0.193)	7 × 10^−22^	0.164 (0.129–0.199)	2 × 10^−16^	0.175 (0.096–0.253)	8 × 10^−5^	0.138 (0.048–0.229)	0.005***	0.008
DGLA	0.034 (0.030–0.039)	2 × 10^−35^	0.035 (0.030–0.040)	2 × 10^−29^	0.032 (0.018–0.046)	5 × 10^−5^	0.028 (0.015–0.041)	3 × 10^−4^	0.368
Arachidonic acid	0.019 (0.001–0.036)	0.038	0.018 (−0.003–0.039)	0.086	0.011 (−0.027–0.049)	0.551	0.034 (−0.035–0.102)	0.311	0.150
6 months									
Linoleic acid	0.127 (0.086–0.167)	4 × 10^−9^	0.120 (0.075–0.165)	7 × 10^−7^	0.179 (0.058–0.300)	0.0212	0.094 (−0.058–0.247)	0.204	0.100
DGLA	0.021 (0.015–0.026)	3 × 10^−12^	0.021 (0.015–0.026)	6 × 10^−17^	0.028 (0.008–0.049)	0.010	0.013 (−0.010–0.035)	0.244	0.643
Arachidonic acid	0.023 (0.003–0.043)	0.024	0.018 (−0.003–0.039)	0.094	0.051 (−0.003–0.106)	0.065	0.018 (−0.095–0.131)	0.735	0.982
12 months									
Linoleic acid	0.070 (0.030–0.109)	0.001	0.072 (0.026–0.118)	0.002	0.099 (−0.008–0.206)	0.069	0.0154 (−0.120–0.151	0.811*	0.059
DGLA	0.013 (0.008–0.019)	6 × 10^−7^	0.013 (0.007–0.020)	9 × 10^−5^	0.016 (−0.001–0.034)	0.065	0.009 (−0.009–0.026)	0.315	0.246
Arachidonic acid	0.034 (0.010–0.059)	0.006	0.030 (0.001–0.058)	0.040	0.034 (−0.026–0.093)	0.252	0.068 (−0.029–0.166)	0.153	0.181
24 months									
Linoleic acid	0.017 (−0.032–0.066)	0.506	0.022 (−0.036–0.080)	0.449	0.007 (−0.139–0.154)	0.918	−0.007 (−0.177–0.162)	0.923	0.152
DGLA	0.00009 (−0.0069–0.0071)	0.980	0.0004 (−0.008–0.008)	0.916	−0.016 (−0.038–0.006)	0.142	0.020 (0.006–0.035)	0.013	0.136
Arachidonic acid	0.036 (0.007–0.065)	0.016	0.029 (−0.002–0.061)	0.069	0.048 (−0.051–0.147)	0.314	0.062 (−0.052–0.177)	0.251	0.338

Data are reported as mean (SD). SD, Standard deviation; FFA, Free fatty acids; DGLA, Dihomo‐γ‐linolenic acid; NDM, Patients without diabetes mellitus; DMH‐NDM, Patients with DM in remission after Roux‐en‐Y gastric bypass surgery (RYGB); DMH‐DMH, Patients with DM not in remission after RYGB. One‐way ANOVA (or Kruskal–Wallis H‐test) is comparing the three patient‐subgroup means (absolute mean, not delta) at either 3, 6, 12, or 24 months; * indicates significant difference (*p* < 0.05) when compared to the NDM group; ** indicates significant difference compared to the DMH‐NDM group; *** indicates significant difference compared to both the NDM group and the DMH – NDM group. Values from Mann–Whitney U‐test and Post hoc p‐values from Tukey and Games‐Howell are not shown in table.

Comparing concentrations from pre‐surgery to 3 months post‐surgery, EPA levels decreased in all three groups whereas DHA only decreased significantly in the groups with diabetes. EPA normalizes after 6 months in the group with persistent diabetes and after 24 months in the other two groups. No significant difference was found after 6 months in the group with persistent diabetes and after 12 months in the group with remission. No difference was found between groups at any time point (Table 5 and Figure 2, for absolute values see supplementary material Table S4).

**TABLE 5:** EPA and DHA delta values (presurgery values minus post‐surgery values) and paired T‐tests grouped according to diabetes status and ANOVA

mmol/L	All		NDM		DMH‐NDM		DMH – DMH		ANOVA
	Mean delta (95% CI)	Paired T‐test *p*‐value	Mean delta (95% CI)	Paired *t*‐test *p*‐value	Mean delta (95% CI)	Paired *t*‐test *p*‐value	Mean delta (95% CI)	Paired *t*‐test *p*‐value	*p*‐value
3 months									
EPA	0.015 (0.013–0.018)	6 × 10^−22^	0.014 (0.011–0.017)	2 × 10^−15^	0.022 (0.013–0.030)	6 × 10^−6^	0.019 (0.010–0.029)	0.004	0.227
DHA	0.009 (0.003–0.014)	0.002	0.006 (−0.0001–0.013)	0.055	0.015 (0.001–0.029)	0.032	0.014 (0.001–0.026)	0.036	0.481
6 months									
EPA	0.014 (0.010–0.017)	6 × 10^−13^	0.012 (0.008–0.016)	1 × 10^−9^	0.023 (0.011–0.034)	0.003	0.011 (−0.001–0.023)	0.091	0.552
DHA	0.011 (0.004–0.020)	0.001	0.006 (−0.001–0.014)	0.098	0.031 (0.012–0.052)	0.003	0.005 (−0.018–0.028)	0.643	0.984
12 months									
EPA	0.010 (0.005–0.013)	4 × 10^−6^	0.088 (0.005–0.013)	4 × 10^−5^	0.017 (0.005–0.028)	0.023	0.00008 (−0.013–0.013)	0.989	0.284
DHA	0.007 (−0.0007–0.015)	0.073	0.005 (−0.003–0.014)	0.248	0.021 (−0.003–0.045)	0.088	0.002 (−0.018–0.022)	0.817	0.480
24 months									
EPA	−0.001 (−0.006–0.003)	0.556	−0.001 (−0.006–0.004)	0.641	0.001 (−0.020–0.017)	0.876	−0.003 (−0.022–0.016)	0.711	0.102
DHA	0.0004 (−0.011–0.010)	0.899	−0.002 (−0.011–0.008)	0.717	0.0003 (−0.052–0.053)	0.989	0.005 (−0.030–0.041)	0.742	0.310

Data are reported as mean (SD). SD, Standard deviation; FFA, Free fatty acids; EPA, Eicosapentaenoic acid; DHA, Docosahexaenoic acid; NDM, Patients without diabetes mellitus; DMH‐NDM, Patients with DM in remission after Roux‐en‐Y gastric bypass surgery (RYGB); DMH‐DMH, Patients with DM not in remission after RYGB. One‐way ANOVA (or Kruskal–Wallis H‐test) is comparing the three patient‐subgroup means (absolute mean, not delta) at either 3, 6, 12, or 24 months.

### 3.3 FFA ratios

We calculated ratios at all time points between the FFAs in the same pathway. The changes in ratios from pre‐surgery to post‐surgery naturally reflected the decrease in concentrations of palmitic acid, stearic acid, linoleic acid, DGLA, and EPA. For instance, the palmitic acid‐oleic acid ratio decreased significantly from pre‐surgery to 3 months after surgery in all groups. After 24 months, ratios did not differ from pre‐surgery levels except for the ratio of linoleic acid to DGLA in the two diabetes groups and the DGLA to the arachidonic acid ratio in the group with diabetes remission, (see supplementary, Table S5, S6, S7).

The FFA ratios did not differ between groups except for the EPA to DHA ratio at 12 months after surgery, where the group with persistent diabetes showed a higher ratio compared to the other two groups (see supplementary, Table S8).

## 4 DISCUSSION

High levels of FFAs are accompanied by defective insulin signaling and beta‐cell dysfunction (Sonnweber et al., 2018), and type 2 diabetes is often accompanied by elevated plasma FFAs (Sobczak & Blindauer, 2019). Interestingly, we found a tendency for total FFA to be lower at all time points in the group of patients with persistent diabetes from 3‐months post‐surgery. Our results contradict results from other studies that have found higher levels of FFA in patients with than without diabetes (Sobczak & Blindauer, 2019). This might be explained by a difference in study populations. In our study, the whole population had severe obesity, something that is known to be strongly correlated with both type 2 diabetes and increased FFA levels. In the mentioned studies, control groups were not BMI‐matched with the diabetes groups (Liyan et al., 2010; Lu et al., 2016; Xiao‐Li & Lei, 2018). In our study, differences in age and gender distribution between the patients with and without diabetes may play a role.

In contrast to a previous metabolomics study that found decreased levels of FFAs in patients with diabetes remission after RYGB, but no decrease in the group with persistent diabetes, we did not find differences between groups (Luo et al., 2016). In all three groups, palmitic acid, stearic acid, linoleic acid, DGLA, and EPA decreased after surgery, while oleic acid, arachidonic acid, and DHA levels stayed unchanged. No significant difference for oleic acid has previously been demonstrated 3 days after RYGB in two other studies that at the same time found significant decreases in both palmitic acid, stearic acid, and linoleic acid (Nemati et al., 2017; Thomas et al., 2016).

The FFAs with decreasing levels are all precursors to oleic acid, arachidonic acid, and DHA, respectively. Thus, the decrease may imply a drift toward oleic acid, arachidonic acid, and DHA, indicating that these FFAs need to be sustained at an adequate level for optimal metabolic function. This agrees with studies showing the vital importance of these three fatty acids in metabolic function in relation to Type 2 diabetes (Das, 2018; Palomer et al., 2018; Shahidi, 2018).

The saturated palmitic acid and the monounsaturated oleic acid are known to affect the mechanisms behind insulin resistance differently (Hu et al., 2011; Palomer et al., 2018). Palmitic acid contributes to the development of insulin resistance, while oleic acid protects cells from the attenuation of the insulin signaling pathway caused by palmitic acid (Hu et al., 2011; Palomer et al., 2018).

The increase from one year to two years post‐surgery in the precursors to oleic acid in the group with diabetes remission may imply that the drift toward oleic acid is no longer needed, while the levels of oleic acid do not get fully “saturated” in the non‐remission diabetes group.

A significant decrease in the palmitic acid to oleic acid ratio was found at 3 months post‐surgery in all three groups. However, the palmitic acid to oleic acid ratios after 24 months was like pre‐surgery levels in all three groups (see supplementary material, Table 5), at a time point where both hepatic and peripheral insulin sensitivity has been markedly improved (Bojsen‐Møller et al., 2014, 2017). Given the influence of palmitic acid and oleic acid on insulin signaling, one might have expected to find an increased oleic acid to the palmitic acid ratio in the group with diabetes remission compared to the group with persistent diabetes after RYGB. In contrast, we find similar ratios in all three groups. Notable, not only improvement in insulin sensitivity is of importance for the remission of diabetes after RYGB, another important denominator for remission is the insulin secretory capacity of the pancreatic beta‐cells (Madsbad et al., 2014).

Our study showed that the level of linoleic acid and DGLA, which are precursors to arachidonic acid, decreases after RYGB, while arachidonic acid levels are maintained after surgery. This may suggest that the intermediate metabolism needs a certain amount of arachidonic acid to function properly. Since type 2 diabetes is associated with chronic inflammation, this is in some ways a surprise. One could argue that lower levels of arachidonic acid should be beneficial due to its pro‐inflammatory metabolites (lipoxins). But maybe the benefits of arachidonic acids anti‐inflammatory metabolites and the fluidity of the cell membranes outweighs the downside of the pro‐inflammatory metabolites. Arachidonic acid metabolism is one of the most complex regulatory systems within the human body and is not fully understood. (Sonnweber et al., 2018).

Linoleic acid levels were significantly lower before surgery in the group of patients with persistent diabetes after RYGB, compared to the group with diabetes remission. This implies, that linoleic acid could possibly be used as a predictive preoperative biomarker for type 2 diabetes remission after RYGB. However, since we do not have information about the dietary intake of FFAs, it is not possible to know whether the pre‐surgery difference in linoleic acid levels is merely an expression of dietary intake or if it serves as a marker for a metabolic difference for example in the downstream pathway of linoleic acid. It is also unknown if the linoleic acid itself is beneficial in regard to diabetes remission after RYGB surgery or if it is simply a marker without causative effect.

EPA and DHA are both precursors to potent anti‐inflammatory resolvins and protectins and are believed to have protective effects against diabetes (Kwon, 2020). Three months post‐surgery, we found markedly significant decreases in EPA levels in all three groups, while a more discrete, but significantly decrease was found in DHA levels in the two groups with diabetes, but not in the group of patients without diabetes. This might indicate that patients with diabetes have lower levels of upstream metabolites in the omega 3 pathway to sustain DHA levels. However, this is not substantiated by the fact that the EPA levels (precursor to DHA) were significantly higher in patients with diabetes remission compared to the non‐diabetes group. Our findings suggest that human metabolism might prioritize to maintain DHA levels to EPA levels.

Delta‐5‐desaturase and delta‐6‐desaturase prefer omega 3 FFAs to omega‐6 FFAs and omega‐6 fatty acids to omega‐9 fatty acids and EPA inhibits the activity of delta‐5‐desaturase and delta‐6‐saturase, which decreases the conversion of linoleic acid to arachidonic acid (Das, 2006).

The decrease of EPA in all groups, therefore, might enable the omega‐6 pathway to sustain arachidonic acid levels.

The fact that linoleic acid and EPA were lower in patients receiving statin treatment suggests that statins might have a lowering effect on linoleic acid‐ and EPA levels. Since linoleic acid levels are lower in the group with persistent diabetes, it is worth considering whether a higher prevalence of statin treatment in this group could explain the lower linoleic acid levels. However, though the prevalence of statin treatment before surgery was higher in the two diabetes groups compared to the group without diabetes, no significant difference was found in the prevalence of statin treatment when comparing the group with persistent diabetes with the group with diabetes remission (see Table 1). There are only 34 and 20 patients in the group with persistent diabetes and the group with diabetes remission, respectively, which is a limitation.

## 5 CONCLUSION

The FFAs with decreasing levels from pre‐surgery to three months post‐surgery are all precursors to oleic acid, arachidonic acid, and DHA, respectively. Thus, the decrease may imply a drift toward oleic acid, arachidonic acid, and DHA, indicating that they need to be sustained at an acceptable level for optimal metabolic function. The increase from one year to two years post‐surgery in the precursors to oleic acid and arachidonic acid in the group with type 2 diabetes remission may imply that the drift toward oleic acid and arachidonic acid is no longer needed, while the levels of oleic acid and arachidonic acid does not get fully “saturated” in the non‐remission diabetes group. The fact that the sum of the measured FFAs is lower in the group with persistent type 2 diabetes compared to the group with type 2 diabetes remission may suggest that they represent two distinct types of type 2 diabetes. It is proposed that linoleic acid could be used as a biomarker to determine the plausibility for type 2 diabetes remission after RYGB.

## CONFLICT OF INTEREST

All authors have has no conflicts of interest.

## Supporting information



Supplementary Material
