# Supplementary material for: Fractionated free fatty acids and their relation to diabetes status after Roux‐en‐Y gastric bypass: A cohort study
Source: Physiol Rep. 2021 Jan 19;9(2):e14708. doi: 10.14814/phy2.14708 (PMC7814490; doi:10.14814/phy2.14708)
Supplement: Supplementary file 1 — Supplementary Material [file PHY2-9-e14708-s001.docx]

Method of analysis - precision

In each batch, the first and last sample was a control sample. We used the control samples from all batches to calculate the variation coefficient (CV) for the 8 FFAs: 20.5 % (palmitic acid), 17.2 % (stearic acid), 18.2 % (oleic acid), 19.1 % (linoleic acid), 12.7 % (DGLA), 16.6 % (arachidonic acid), 18.0 % (EPA), 18.0 % (DHA).

All samples from the same patient were analyzed right after each other in one batch. Thus, the relatively high CV% in the standards does not influence comparing of samples from the same patient, since they are measured using the same calibration curve.

All NDM DMH-NDM DMH – DMH ANOVA

|  | N | Mean (95% CI) | N | Mean (95% CI) | N | Mean (95% CI) | N | Mean (95% CI) | P-value |
| --- | --- | --- | --- | --- | --- | --- | --- | --- | --- |
| 3 months |  |  |  |  |  |  |  |  |  |
| Weight (kg) | 153 | 103.1 (100.8-106.0) | 114 | 103.3 (99.8-106.7) | 25 | 104.7 (96.8-112.6) | 14 | 98.4 (88.3-108-6) | 0.587 |
| BMI (kg/m^2^) | 153 | 34.9 (34.1-35.7) | 114 | 35.3 (34.4-36.3) | 25 | 34.5 (32.3-36.7) | 14 | 32.4 (30.5-34.2) | 0.100 |
| Weight loss (kg) | 153 | 22.6 (21.5-23.8) | 114 | 22.6 (21.3-23.9) | 25 | 22.2 (19.2-25.2) | 14 | 23.4 (19.3-27.5) | 0.882 |
| Weight loss (%) | 153 | 17.2 (17.1-18.6) | 114 | 17.9 (17.1-18.7) | 25 | 17.6 (15.4-19.7) | 14 | 19.1 (16.7-21.4) | 0.578 |
| 6 months |  |  |  |  |  |  |  |  |  |
| Weight (kg) | 139 | 95.0 (91.8-97.9) | 102 | 95.5 (91.9-99.0) | 22 | 93.9 (85.7-102.0) | 15 | 92.2 (82.4-101.9) | 0.774 |
| BMI (kg/m^2^) | 139 | 32.2 (31.4-33.0) | 102 | 32.7 (31.7-33.6) | 22 | 31.3 (29.0-33.6) | 15 | 30.4 (28.5-32.3) | 0.148 |
| Weight loss (kg) | 139 | 30.7 (29.2-32.3) | 102 | 31.2 (29.4-33.1) | 22 | 30.2 (25.7-34.7) | 15 | 28.2 (24.1-32.3) | 0.491 |
| Weight loss (%) | 139 | 24.4 (23.4-25.4) | 102 | 24.5 (23.4-25.6) | 22 | 24.4 (21.2-27.7) | 15 | 23.4 (21.0 -25.8) | 0.777 |
| 12 months |  |  |  |  |  |  |  |  |  |
| Weight (kg) | 109 | 87.1 (83.6-90.0) | 81 | 85.8 (82.1-89.5) | 14 | 91.7 (82.0-101.4) | 14 | 87.6 (76.6-98.7) | 0.487 |
| BMI (kg/m^2^) | 109 | 29.8 (28.9-30.6) | 81 | 29.7 (28.7-30.8) | 14 | 30.6 (27.8 -33.5) | 14 | 29.0 (26.8 -31.3) | 0.668 |
| Weight loss (kg) | 109 | 35.6 (33.4-37.9) | 81 | 36.8 (34.2-39.4) | 14 | 32.8 (25.0-40.7) | 14 | 31.7 (26.9-36.6) | 0.218 |
| Weight loss (%) | 109 | 29.1 (27.5-30.6) | 81 | 29.9 (28.2-31.7) | 14 | 26.3 (20.7-31.8) | 14 | 26.7 (23.4-30.1) | 0.146 |
| 24 months |  |  |  |  |  |  |  | Mean (min-max) |  |
| Weight (kg) | 35 | 85.4 (79.7-91.0) | 26 | 82.7 (77.0-88.3) | 7 | 99.9 (83.3-116.6)* | 2 | 69.6 (60.7-78.4) | 0.013 |
| BMI (kg/m^2^) | 35 | 29.4 (27.9-31.0) | 26 | 28.8 (27.1-30.5) | 7 | 32.5 (28.2-36.9) | 2 | 26.8 (25.9-27.7) | 0.094 |
| Weight loss (kg) | 35 | 36.6 (31.6-41.6) | 26 | 38.4(32.4-44.4) | 7 | 30.1 (18.1-42.0) | 2 | 36.3 (24.0-48.6) | 0.421 |
| Weight loss (%) | 35 | 29.8 (26.5-33.1) | 26 | 31.3 (27.5-35.0) | 7 | 23.5 (13.8-33.1) | 2 | 33.3 (28.3-38.3) | 0.146 |

**Table S1:** Data are reported as mean (95% CI), except for 24 months postoperative in the DMH-DMH group, where the numbers in parenthesis resembles minimum and maximum values (N = 2). NDM, Patients without diabetes mellitus (DM); DMH-NDM, Patients with DM in remission after Roux-en-Y gastric bypass surgery (RYGB); DMH-DMH, Patients with DM not in remission after RYGB. The p-value from One-way ANOVA compares the three patient-subgroup means; * indicates significant difference (p < 0,05) when compared to the NDM group. No significant differences were found between the two diabetes subgroups.

|  | Female Mean (SD) | Male Mean (SD) | Unpaired t-test p-value |
| --- | --- | --- | --- |
| **Palmitic acid** |  |  |  |
| All patients (F142 /M64) | 1.773 (0.46) | 1.673 (0.50) | 0.163 |
| NDM (F115/M37) | 1.764 (0.44) | 1.669 (0.49) | 0.270 |
| DMH-NDM (F18/M16) | 1.833 (0.58) | 1.833 (0.53) | 0.998 |
| DMH-DMH (F9/M11) | 1.458 (0.43) | 1.458 (0.43) | 0.141 |
| **Stearic acid** |  |  |  |
| All patients (F142 /M65) | 0.525 (0.12) | 0.535 (0.12) | 0.602 |
| NDM (F115/M37) | 0.525 (0.12) | 0.551 (0.12) | 0.272 |
| DMH-NDM (F18/M16) | 0.514 (0.12) | 0.551 (0.14) | 0.410 |
| DMH-DMH (F9/M11) | 0.545 (0.10) | 0.457 (0.10) | 0.053 |
| **Oleic acid** |  |  |  |
| All patients (F142 /M65) | 0.881 (0.25) | 0.839 (0.22) | 0.250 |
| NDM (F115/M37) | 0.872 (0.24) | 0.817 (0.20) | 0.200 |
| DMH-NDM (F18/M16) | 0.935 (0.31) | 0.949 (0.15) | 0.862 |
| DMH-DMH (F9/M11) | 0.879 (0.33) | 0.752 (0.31) | 0.387 |
| **Linoleic acid** |  |  |  |
| All patients (F142 /M65) | 0.887 (0.27) | 0.792 (0.24) | 0.018 |
| NDM (F115/M37) | 0.889 (0.25) | 0.834 (0.24) | 0.246 |
| DMH-NDM (F18/M16) | 0.917 (0.39) | 0.823 (0.23) | 0.410 |
| DMH-DMH (F9/M11) | 0.802 (0.28) | 0.603 (0.18) | 0.070 |
| **DGLA** |  |  |  |
| All patients (F142 /M65) | 0.100 (0.033) | 0.090 (0.033) | 0.083 |
| NDM (F115/M37) | 0.100 (0.042) | 0.095 (0.031) | 0.538 |
| DMH-NDM (F18/M16) | 0.108 (0.056) | 0.085 (0.039) | 0.174 |
| DMH-DMH (F9/M11) | 0.091 (0.029) | 0.078 (0.026) | 0.303 |
| **Arachidonic acid** |  |  |  |
| All patients (F142 /M65) | 0.474 (0.17) | 0.463 (0.17) | 0.677 |
| NDM (F115/M37) | 0.472 (0.17) | 0.044 (0.15) | 0.380 |
| DMH-NDM (F18/M16) | 0.501 (0.21) | 0.510 (0.20) | 0.899 |
| DMH-DMH (F9/M11) | 0.447 (0.18) | 0.459 (0.17) | 0.879 |
| **EPA** |  |  |  |
| All patients (F142 /M65) | 0.042 (0.024) | 0.043 (0.025) | 0.878 |
| NDM (F115/M37) | 0.041 (0.024) | 0.035 (0.016) | 0.114 |
| DMH-NDM (F18/M16) | 0.043 (0.023) | 0.063 (0.034) | 0.047 |
| DMH-DMH (F9/M11) | 0.055 (0.028) | 0.042 (0.018) | 0.230 |
| **DHA** |  |  |  |
| All patients (F142 /M65) | 0.128 (0.050) | 0.118 (0.048) | 0.185 |
| NDM (F115/M37) | 0.126 (0.047) | 0.112 (0.044) | 0.102 |
| DMH-NDM (F18/M16) | 0.134 (0.060) | 0.142 (0.056) | 0.687 |
| DMH-DMH (F9/M11) | 0.141 (0.070) | 0.105 (0.038) | 0.164 |

**Table S2**: Unpaired t-test comparing pre-surgery FFA levels between genders in all patients and in the three subgroups. FXX/MXX: F = Female. M = Male and the numbers following are the number of female or male patients in the group**.**

| Ratios | Statin treatment Mean (SD) | No treatment Mean (SD) | Unpaired t-test p-value |
| --- | --- | --- | --- |
| **Palmitic acid** |  |  |  |
| All patients (S56/N145) | 1.760 (0.53) | 1.725 (0.45) | 0.604 |
| NDM (S20/N128) | 1.855 (0.52) | 1.718 (0.44) | 0.210 |
| DMH-NDM (S21/N12) | 1.759 (0.57) | 1.914 (0.500) | 0.441 |
| DMH-DMH (S15/N5) | 1.636 (0.48) | 1.481 (0.44) | 0.531 |
| **Stearic acid** |  |  |  |
| All patients (S56/N146) | 0.533 (0.12) | 0.524 (0.12) | 0.601 |
| NDM (S20/N129) | 0.571 (0.11) | 0.524 (0.12) | 0.112 |
| DMH-NDM (F18/M16) | 0.523 (0.13) | 0.536 (0.13) | 0.781 |
| DMH-DMH (S15/N5) | 0.500 (0.10) | 0.485 (0.11) | 0.783 |
| **Oleic acid** |  |  |  |
| All patients (S56/N146) | 0.869 (0.25) | 0.862 (0.24) | 0.649 |
| NDM (S20/N129) | 0.858 (0.21) | 0.855 (0.23) | 0.960 |
| DMH-NDM (S21/N12) | 0.909 (0.20) | 0.976 (0.31) | 0.453 |
| DMH-DMH (S15/N5) | 0.828 (0.34) | 0.753 (0.25) | 0.661 |
| **Linoleic acid** |  |  |  |
| All patients (S56/N146) | 0.755 (0.25) | 0.892 (0.27) | 0.001 |
| NDM (S20/N129) | 0.804 (0.25) | 0.882 (0.25) | 0.186 |
| DMH-NDM (S21/N12) | 0.781 (0.25) | 1.024 (0.40) | 0.040 |
| DMH-DMH (S15/N5) | 0.652 (0.22) | 0.814 (0.31) | 0.211 |
| **DGLA** |  |  |  |
| All patients (S56/N146) | 0.093 (0.041) | 0.098 (0.041) | 0.303 |
| NDM (S20/N129) | 0.107 (0.047) | 0.097 (0.039) | 0.278 |
| DMH-NDM (S21/N12) | 0.088 (0.042) | 0.115 (0.059) | 0.134 |
| DMH-DMH (S15/N5) | 0.081 (0.023) | 0.093 (0.039) | 0.395 |
| **Arachidonic acid** |  |  |  |
| All patients (S56/N146) | 0.488 (0.17) | 0.462 (0.17) | 0.332 |
| NDM (S20/N129) | 0.476 (0.15) | 0.459 (0.17) | 0.679 |
| DMH-NDM (S21/N12) | 0.498 (0.18) | 0.531 (0.24) | 0.569 |
| DMH-DMH (S15/N5) | 0.489 (0.17) | 0.349 (0.12) | 0.113 |
| **EPA** |  |  |  |
| All patients (S56/N146) | 0.051 (0.027) | 0.039 (0.022) | 0.001 |
| NDM (S20/N129) | 0.041 (0.022) | 0.039 (0.022) | 0.672 |
| DMH-NDM (S21/N12) | 0.060 (0.033) | 0.041 (0.021) | 0.090 |
| DMH-DMH (S15/N5) | 0.052 (0.023) | 0.035 (0.018) | 0.137 |
| **DHA** |  |  |  |
| All patients (S56/N146) | 0.131 (0.053) | 0.123 (0.048) | 0.270 |
| NDM (S20/N129) | 0.126 (0.050) | 0.122 (0.046) | 0.723 |
| DMH-NDM (S21/N12) | 0.138 (0.057) | 0.144 (0.058) | 0.761 |
| DMH-DMH (S15/N5) | 0.130 (0.055) | 0.097 (0.059) | 0.262 |

**Table S3:** Unpaired t-test comparing pre-surgery FFA levels between patients in statin treatment and patients with no lipid lowering treatment in all patients and in the three subgroups. SXX/NXX: S = Statin treatment. N = No treatment and the numbers following are the number of female or male patients in the group. SD, Standard deviation; DGLA, Dihomo-γ-linolenic acid; EPA, Eicosapentaenoic acid; DHA, Docosahexaenoic acid; NDM, Patients without diabetes mellitus; DMH-NDM, Patients with DM in remission after Roux-en-Y gastric bypass surgery (RYGB); DMH-DMH, patients with DM not in remission after RYGB.

| mmol/L | Pre-surgery Mean (SD) | 3 months Post-surgery Mean (SD) | 6 months Post-surgery Mean (SD) | 12 months Post-surgery Mean (SD) | 24 months Post-surgery Mean (SD) |
| --- | --- | --- | --- | --- | --- |
| All patients |  |  |  |  |  |
| Total FFA | 4.73 (1.14) | 4.13 (0.89) | 4.20 (0.88) | 4.25 (0.82) | 4.42 (1.06) |
| Palmitic acid | 1.74 (0.47) | 1.49 (0.36) | 1.47 (0.34) | 1.47 (0.33) | 1.54 (0.42) |
| Stearic acid | 0.528 (0.12) | 0.425 (0.093) | 0.457 (0.096) | 0.481 (0.091) | 0.510 (0.12) |
| Oleic acid | 0.868 (0.24) | 0.864 (0.21) | 0.858 (0.21) | 0.857 (0.20) | 0.851 (0.24) |
| Linoleic acid | 0.857 (0.27) | 0.694 (0.21) | 0.738 (0.22) | 0.776 (0.22) | 0.816 (0.22) |
| DGLA | 0.0968 (0.040) | 0.0627 (0.024) | 0.0754 (0.031) | 0.0811 (0.029) | 0.0951 (0.043) |
| Arachidonic acid | 0.470 (0.17) | 0.452 (0.15) | 0.445 (0.13) | 0.436 (0.14) | 0.436 (0.15) |
| EPA | 0.0425 (0.024) | 0.0270 (0.015) | 0.030 (0.016) | 0.0337 (0.017) | 0.0433 (0.026) |
| DHA | 0.125 (0.049) | 0.117 (0.043) | 0.119 (0.043) | 0.117 (0.043) | 0.125 (0.052) |
| NDM |  |  |  |  |  |
| Total FFA | 4.73 (1.09) | 4.16 (0.87) | 4.20 (0.81) | 4.31 (0.81) | 4.35 (0.94) |
| Palmitic acid | 1.74 (0.46) | 1.50 (0.35) | 1.46 (0.32) | 1.49 (0.33) | 1.52 (0.38) |
| Stearic acid | 0.533 (0.12) | 0.423 (0.090) | 0.459 (0.093) | 0.487 (0.092) | 0.502 (0.10) |
| Oleic acid | 0.941 (0.23) | 0.861 (0.20) | 0.853 (0.22) | 0.864 (0.19) | 0.836 (0.20) |
| Linoleic acid | 0.875 (0.25) | 0.711 (0.22) | 0.751 (0.22) | 0.794 (0.23) | 0.816 (0.22) |
| DGLA | 0.0970 (0.039) | 0.0630 (0.024) | 0.0755 (0.032) | 0.0833 (0.030) | 0.0968 (0.046) |
| Arachidonic acid | 0.465 (0.16) | 0.447 (0.13) | 0.445 (0.13) | 0.436 (0.12) | 0.426 (0.13) |
| EPA | 0.0400 (0.023) | 0.0260 (0.014) | 0.0300 (0.017) | 0.0331 (0.016) | 0.0398 (0.022) |
| DHA | 0.123 (0.046) | 0.116 (0.040) | 0.119 (0.044) | 0.119 (0.042) | 0.121 (0.044) |
| DMH-NDM |  |  |  |  |  |
| Total FFA | 4.97 (1.32) | 4.34 (1.04) | 4.40 (1.04) | 4.33 (0.90) | 5.09 (1.53) |
| Palmitic acid | 1.83 (0.55) | 1.55 (0.39) | 1.60 (0.46) | 1.46 (0.31) | 1.83 (0.58) |
| Stearic acid | 0.531 (0.13) | 0.449 (0.11) | 0.464 (0.11) | 0.472 (0.080) | 0.595 (0.16) |
| Oleic acid | 0.873 (0.25) | 0.928 (0.24) | 0.925 (0.22) | 0.916 (0.27) | 0.986 (0.39) |
| Linoleic acid | 0.873 (0.33) | 0.698 (0.22) | 0.743 (0.24) | 0.779 (0.22) | 0.888 (0.28) |
| DGLA | 0.0837 (0.049) | 0.0649 (0.027) | 0.0791 (0.026) | 0.0769 (0.024) | 0.104 (0.033) |
| Arachidonic acid | 0.505 (0.20) | 0.494 (0.21) | 0.450 (0.15) | 0.475 (0.20) | 0.491 (0.24) |
| EPA | 0.0532 (0.030) | 0.0307 (0.014) | 0.0287 (0.012) | 0.030 (0.011) | 0.0539 (0.032) |
| DHA | 0.138 (0.058) | 0.123 (0.047) | 0.117 (0.039) | 0.117 (0.039) | 0.144 (0.078) |
| DMH-DMH |  |  |  |  |  |
| Total FFA | 4.30 (1.13) | 3.66 (0.61) | 3.84 (0.66) | 3.78 (0.69) | 3.90 (0.53) |
| Palmitic acid | 1.59 (0.46) | 1.33 (0.33) | 1.34 (0.31) | 1.34 (0.33) | 1.32 (0.28) |
| Stearic acid | 0.496 (0.10) | 0.394 (0.076) | 0.428 (0.10) | 0.448 (0.093) | 0.444 (0.071) |
| Oleic acid | 0.809 (0.32) | 0.773 (0.16) | 0.787 (0.13) | 0.736 (0.13) | 0.757 (0.18) |
| Linoleic acid | 0.692 (0.25) | 0.554 (0.13) | 0.612 (0.14) | 0.647 (0.18) | 0.711 (0.011) |
| DGLA | 0.0968 (0.028) | 0.0558 (0.016) | 0.0689 (0.025) | 0.0708 (0.024) | 0.0708 (0.018) |
| Arachidonic acid | 0.454 (0.17) | 0.420 (0.12) | 0.444 (0.14) | 0.387 (0.11) | 0.425 (0.10) |
| EPA | 0.048 (0.023) | 0.0291 (0.026) | 0.0347 (0.020) | 0.0419 (0.028) | 0.0519 (0.039) |
| DHA | 0.123 (0.056) | 0.108 (0.058) | 0.118 (0.057) | 0.104 (0.053) | 0.127 (0.059) |

**Table S4:** Data are reported as mean (SD). SD, Standard deviation; DGLA, Dihomo-γ-linolenic acid; EPA, Eicosapentaenoic acid; DHA, Docosahexaenoic acid; NDM, Patients without diabetes mellitus; DMH-NDM, Patients with DM in remission after Roux-en-Y gastric bypass surgery (RYGB); DMH-DMH, patients with DM not in remission after RYGB.

All NDM DMH-NDM DMH-DMH

| Ratios | Mean delta (95% CI) | Paired t-test p-value | Mean delta (95% CI) | Paired t-test p-value | Mean delta (95% CI) | Paired T-test | Mean delta (95% CI) | Paired t-test p-value |
| --- | --- | --- | --- | --- | --- | --- | --- | --- |
| 3 months |  |  |  |  |  |  |  |  |
| Palmitic / stearic | -0.237 (-0.350—0.123) | 5.6x10^-5^ | -0.296 (-0.426-(-0.166)) | 1.4x10^-5^ | -0.031 (-0.278-0.217) | 0.804 | -0.135 (-0.624-0.354) | 0.570 |
| Palmitic/ oleic | 0.293 (0.233-0.353) | 2.1x10^-18^ | 0.297 (0.228-0.367) | 2.1x 0^-14^ | 0.270 (0.150-0.390) | 6.5x10^-5^ | 0.300 (0.023-0.576) | 0.035 |
| Stearic/ oleic | 1.127 (0.102-0.152) | 1.3x10^-19^ | 0.134 (0.103-0.165) | 1.3x19^-14^ | 0.088 (0.048-0.128) | 9.6x10^-5^ | 0.136 (0.057-0.214) | 0.002 |
| 6 months |  |  |  |  |  |  |  |  |
| Palmitic / stearic | 0.052 (-0.064-0.168) | 0.380 | 0.037 (-0.090-0.163) | 0.565 | 0.014 (-0.350-0.377) | 0.938 | 0.246 (-0.274-0.767) | 0.322 |
| Palmitic/ oleic | 0.306 (0.244-0.367) | 1.1x10^-17^ | 0.317 (0.246-0.388) | 1.4x10^-14^ | 0.270 (0.124-0.417) | 0.001 | 0.267 (-0.022-0.576) | 0.067 |
| Stearic/ oleic | 0.094 (0.067-0.121) | 1.0x10^-10^ | 0.101 (0.069-0.133) | 5.7x10^-9^ | 0.075 (0.012-0.139) | 0.022 | 0.067 (-0.027-0.161) | 0.144 |
| 12 months |  |  |  |  |  |  |  |  |
| Palmitic / stearic | 0.202 (0.060-0.318) | 0.004 | 0.203 (0.052-0.355) | 0.009 | 0.161 (-0.200-0.522) | 0.361 | 0.245 (-0.391-0.880) | 0.423 |
| Palmitic/ oleic | 0.317 (0.241-0.394) | 1.5x10^-13^ | 0.345 (0.254-0.435) | 1.8x10^-11^ | 0.285 (0.139-0.431) | 0.001 | 0.169 (-0.123-0.461) | 0.235 |
| Stearic/ oleic | 0.068 (0.037-0.100) | 2.7x10^-5^ | 0.076 (0.040-0.112) | 5.7x10^-5^ | 0.065 (-0.019-0.149) | 0.119 | 0.018 (0.093-0.129) | 0.733 |
| 24 months |  |  |  |  |  |  |  |  |
| Palmitic / stearic | 0.181 (0.005-0.356) | 0.043 | 0.121 (-0.055-0.298) | 0.175 | 0.196 (-0.265-0.656) | 0.375 | 0.564 (-0.490-1.618) | 0.257 |
| Palmitic/ oleic | 0.154 (0.082-0.227) | 5.3x10^-5^ | 0.181 (0.096-0.265) | 6.4 x 10^-5^ | -0.030 (-0.201-0.140) | 0.709 | 0.234 (-0.027-0.495) | 0.073 |
| Stearic/ oleic | 0.021 (-0.016-0.057) | 0.262 | 0.037 (-0.006-0.079) | 0.088 | -0.040 (-0.127-0.047) | 0.337 | -0.005 (-0.159-0.139) | 0.940 |

**Tabel S5:** Paired t-test comparing FFA ratios between pre- and post-surgery. Data are reported as mean (SD). SD, Standard deviation; NDM, patients without diabetes mellitus (DM); DMH-NDM, patients with DM in remission after Roux-en-Y gastric bypass surgery (RYGB); DMH-DMH, patients with DM not in remission after RYGB.

All NDM DMH-NDM DMH-DMH

| Ratios | Mean delta (95% CI) | Paired t-test p-value | Mean delta (95% CI) | Paired t-test p-value | Mean delta (95% CI) | Paired t-test | Mean delta (95% CI) | Paired t-test p-value |
| --- | --- | --- | --- | --- | --- | --- | --- | --- |
| 3 months |  |  |  |  |  |  |  |  |
| Linoleic/ DGLA | -2.210 (-2.692-( 1.728)) | 1.1x10^-16^ | -2.361 (-2.949-1.772) | 4.5x10^-13^ | -1.752 (-2.881-(-0.622)) | 0.003 | -1.841 (-3.047-(-0.626)) | 0.005 |
| DGLA/ arachidonic | 0.068 (0.060-0.075) | 7.3x10^-43^ | 0.072 (0.063-0.081) | 2.1x10^-34^ | 0.055 (0.038-0.073) | 3.6x10^-7^ | 0.054 (0.028-0.081) | 4x10^-4^ |
| 6 months |  |  |  |  |  |  |  |  |
| Linoleic/ DGLA | -0.820 (-1.382-(-0.259) | 0.004 | -0.859 (-1.536-(0.182)) | 0.013 | -0.713 (-0.915-0.490) | 0.232 | -0.663 (-2.583-1.261) | 0.467 |
| DGLA/ arachidonic | 0.038 (0.029-0.047) | 6.8x10^-14^ | 0.041 (0.030-0.052) | 3.8x10^-12^ | 0.028 (0.003-0.053) | 0.028 | 0.026 (-0.005-0.568) | 0.091 |
| 12 months |  |  |  |  |  |  |  |  |
| Linoleic/ DGLA | -0.377 (-0.970-0.215) | 0.211 | -0.332 (-1.055-0.390) | 0.364 | -0.245 (-0.178-1.292) | 0.741 | -0.852 (-2.274-1.261) | 0.219 |
| DGLA/ arachidonic | 0.014 (0.004-0.025) | 0.007 | 0.016 (0.004-0.029) | 0.013 | 0.019 (0.0003-0.037) | 0.047 | -0.004 (-0.037-0.030) | 0.833 |
| 24 months |  |  |  |  |  |  |  |  |
| Linoleic/ DGLA | 0.0171 (-0.582-0.925) | 0.653 | -0.223 (-0.685-1.130) | 0.626 | 1.889 (0.078-3.700) | 0.042 | 0.054 (0.028-0.081) | 2x10^-4^ |
| DGLA/ arachidonic | -0.015 (-0.029-0.001) | 0.032 | -0.014 (-0.030-0.002) | 0.090 | -0.057 (-0.088-(-0.025)) | 0.002 | 0.032 (-0.010-0.074) | 0.117 |

**Tabel S6:** Paired t-test comparing FFA ratios between pre- and post-surgery. Data are reported as mean (SD). SD, standard deviation, DGLA, Dihomo-γ-linolenic acid; NDM, Patients without diabetes mellitus (DM); DMH-NDM Patients with DM in remission after Roux-en-Y gastric bypass surgery (RYGB); DMH-DMH, Ppatients with DM not in remission after RYGB.

All NDM DMH-NDM DMH-DMH

| Ratios | Mean delta (95% CI) | Paired t-test p-value | Mean delta (95% CI) | Paired t-test p-value | Mean delta (95% CI) | Paired t-test p-value | Mean delta (95% CI) | Paired t-test p-value |
| --- | --- | --- | --- | --- | --- | --- | --- | --- |
| 3 months |  |  |  |  |  |  |  |  |
| EPA/DHA | 0.111 (0.094-0.127) | 3.3x10^-29^ | 0.105 (0.085-0.124) | 6.5x10^-20^ | 0.121 (0.078-0.164) | 2.0x10^-6^ | 0.137 (0.091-0.183) | 6x10^-6^ |
| 6 months |  |  |  |  |  |  |  |  |
| EPA/DHA | 0.089 (0.069-0.110) | 1.4x10^-14^ | 0.090 (0.066-0.114) | 3.4x10^-11^ | 0.094 (0.038-0.152) | 0.002 | 0.073 (0.013-0.133) | 0.021 |
| 12 months |  |  |  |  |  |  |  |  |
| EPA/DHA | 0.050 (0.025-0.075) | 9.8x10^-5^ | 0.057 (0.029-0.086) | 1.2x10^-4^ | 0.074 (0.023-0.125) | 0.007 | -0.030 (-0.127-0.067) | 0.517 |
| 24 months |  |  |  |  |  |  |  |  |
| EPA/DHA | 0.001 (-0.028-0.031) | 0.930 | 0.002 (-0.031-0.037) | 0.888 | -0.007 (-0.086-0.072) | 0.853 | 0.005 (-0.107-0.117) | 0.920 |

**Tabel S7:** Paired t-test comparing FFA ratios between pre- and post-surgery. Data are reported as mean (SD). SD, standard deviation; EPA, Eicosapentaenoic acid; DHA, Docosahexaenoic acid; NDM, patients without diabetes mellitus (DM); DMH-NDM, patients with DM in remission after Roux-en-Y gastric bypass surgery (RYGB); DMH-DM, patients with DM not in remission after RYGB.

| Ratios | Pre surgery | 3 months | 6 months | 12 months | 24 months |
| --- | --- | --- | --- | --- | --- |
| Palmitic / stearic | 0.521 | 0.317 | 0.198 | 0.914 | 0.959 |
| Stearic/ oleic | 0.132 | 0.707 | 0.339 | 0.213 | 0.851 |
| Linoleic/ DGLA | 0.351 | 0.167 | 0.428 | 0.708 | 0.461 |
| DGLA/ arachidonic | 0.115 | 0.860 | 0.381 | 0.444 | 0.067 |
| EPA/DHA | 0.015 | 0.009 | 0.111 | 8x10^-5^ ** | 0.420 |

**Table S8:** P-values from One-way ANOVA (or Kruskal-Wallis H-test) comparing the three patient-subgroup means at either pre-surgery 3-, 6-, 12- and 24 months. DGLA, Dihomo-γ-linolenic acid; EPA, Eicosapentaenoic acid; DHA, Docosahexaenoic acid. * indicates significant difference (p < 0.05) when comparing the NMD group to the DMH-DMH group; ** indicates significant difference when comparing the DMH-DMH group to both the NMD- and the DMH-NMD group; Post hoc p-values from Tukey and Games-Howell are not shown in table. P-values below 0.05 without a * did not reach significance with the post hoc test.
